# A participatory and capacity-building approach to healthy eating and physical activity – SCIP-school: a 2-year controlled trial

**DOI:** 10.1186/1479-5868-9-145

**Published:** 2012-12-17

**Authors:** Liselotte Schäfer Elinder, Nelleke Heinemans, Jan Hagberg, Anna-Karin Quetel, Maria Hagströmer

**Affiliations:** 1 Division of Social Medicine, Department of Public Health Sciences, Karolinska Institutet, 171 76, Stockholm, Sweden; 2 Division of Occupational and Environmental Medicine, Institute of Environmental Medicine, Karolinska Institutet, 171 77, Stockholm, Sweden; 3 Division of Physiotherapy, Department of Neurobiology, Care Sciences and Society, Karolinska Institutet, Box 23100, 141 83, Huddinge, Sweden

**Keywords:** Children, Eating habits, Exercise, Fidelity, Health promotion, Obesity prevention, Process evaluation, Quasi-experimental study, Self-esteem

## Abstract

**Background:**

Schools can be effective settings for improving eating habits and physical activity, whereas it is more difficult to prevent obesity. A key challenge is the “implementation gap”. Trade-off must be made between expert-driven programmes on the one hand and contextual relevance, flexibility, participation and capacity building on the other. The aim of the Stockholm County Implementation Programme was to improve eating habits, physical activity, self-esteem, and promote a healthy body weight in children aged 6–16 years. We describe the programme, intervention fidelity, impacts and outcomes after two years of intervention.

**Methods:**

Nine out of 18 schools in a middle-class municipality in Sweden agreed to participate whereas the other nine schools served as the comparison group (quasi-experimental study). Tailored action plans were developed by school health teams on the basis of a self-assessment questionnaire called KEY assessing strengths and weaknesses of each school’s health practices and environments. Process evaluation was carried out by the research staff. Impacts at school level were assessed yearly by the KEY. Outcome measures at student level were anthropometry (measured), and health behaviours assessed by a questionnaire, at baseline and after 2 years. All children in grade 2, 4 and 7 were invited to participate (n=1359) of which 59.8% consented. The effect of the intervention on health behaviours, self-esteem, weight status and BMIsds was evaluated by unilevel and multilevel regression analysis adjusted for gender and baseline values.

**Results:**

Programme fidelity was high demonstrating feasibility, but fidelity to school action plans was only 48% after two years. Positive and significant (p<.05) impacts were noted in school health practices and environments after 2 years. At student level no significant intervention effects were seen for the main outcomes.

**Conclusions:**

School staff has the capacity to create their own solutions and make changes at school level on the basis of self-assessment and facilitation by external agents. However these changes were challenging to sustain over time and had little impact on student behaviours or weight status. Better student outcomes could probably be attained by a more focused and evidence-based approach with stepwise implementation of action plans.

## Background

Children and youth in Sweden on average have a high intake of energy-dense foods and sweetened beverages and a low intake of fruit and vegetables relative to the Swedish nutrition recommendations [1]. In addition, the fitness of adolescents has been on the decline for decades [2], probably as a result of decreasing physical activity, leading to energy imbalance and rising body weight. The prevalence of obesity has been rising during the last two decades [3,4] although a stabilisation seems to have occurred among children in Sweden at a level around 3% in 8–10 year olds [5-8]. However, socioeconomic differences in obesity prevalence prevail [6].

There are strong links between children’s health, health behaviours and academic achievement [9], which is a strong argument for health interventions in schools. Furthermore, there is mounting evidence that schools are effective settings for promoting healthy eating habits and physical activity in children and youth. It has been suggested that comprehensive interventions are the most successful, combining health education, provision of supportive social and physical environments with psycho-social support [10-13]. Although there is insufficient evidence for any particular programme that effectively prevents obesity, there is now support for the hypothesis that obesity prevention in schools can be effective and does not cause adverse outcomes or increased health inequalities [14]. The following measures have been included in beneficial programmes: Health education, physical education classes, food supply, supportive environments for healthy diets and physical activity, training of staff and capacity building, and parental involvement. There is also evidence to suggest that girls are more responsive to educational strategies and boys to environmental changes [15,16], indicating that both types of strategies should be employed.

Concerns have been raised that too strong emphasis on obesity prevention could provoke unwanted weight-control measures and eating disorders in adolescents [17]. Therefore, the expressed focus of school-based interventions should be on healthful eating and physical activity behaviours, instead of dieting and weight loss. This also avoids stigmatisation of already overweight children. It has been suggested that programmes addressing body weight should also include measures to develop self-esteem and psychosocial well-being in adolescents, and measures to improve body image [18].

A key challenge of school health promotion programmes is the “implementation gap”, meaning that effective programmes are often not implemented correctly or sustained in the school’s reality [19,20]. Intervention fidelity, defined as the extent to which a programme adheres to its programme theory [21], is less often evaluated, but is crucial to understanding the outcome at an individual level [22-24]. With regard to sustainability, a prerequisite is the presence of local capacity, which is often insufficient in schools and needs to be built through external support [19]. Capacity has been described as the ability and motivation to identify, prioritise, plan, implement, evaluate and sustain health interventions [25]. Furthermore, involving stakeholders in programme design, implementation and evaluation is crucial to the success of interventions and to sustainability [20,26,27]. It has been suggested that benefits of a health intervention as perceived by users are of higher importance for sustainability than effectiveness is [26]. In an effort to obtain both effectiveness and sustainability, trade-offs must be made between the need for an effective programme with predefined, evidence-based and fully implemented components on the one hand, and a contextually relevant programme which builds on local needs and opportunities and has a participatory approach, on the other.

The Stockholm County Implementation Programme in school (SCIP-school) is a model project as part of the Stockholm County Overweight and Obesity Action Plan 2004–2010, revised 2010–2013 [28]. The aim of the programme was to improve eating habits, physical activity, self-esteem, and promote a healthy body weight in children aged 6–16 years. Since capacity-building and sustainability were in focus, a participatory and flexible approach was chosen, which would allow the schools to create their own solutions to healthy eating and physical activity within a systematic framework and based on research. The purpose of this paper is to describe the SCIP-school programme, intervention fidelity, impacts at school level and outcomes at student level after two years.

## Methods

### Study design, schools and recruitment

The SCIP-school programme was implemented in the middle-class municipality of Österåker with 39,000 inhabitants in Stockholm County on the request by municipal representatives. The socioeconomic status of inhabitants is slightly above the Swedish average, as is life expectancy and level of employment, whereas proportion of citizens with a non-Swedish background, and child poverty is lower (Table 1). The design of the study was quasi-experimental with nine schools out of 18 possible agreeing to participate in the programme after presentation to headmasters. Children in the other nine schools that did not sign up to the programme served as the comparison group. Each participating school was visited by the research team together with a municipality representative before the project began, to establish the programme locally. A steering committee was formed with the research team, three representatives from the municipality, the local authority chief nurse and chief catering manager. The latter was involved since school meals play a central role in the school nutrition environment in Sweden, where it is served for free to all children.

**Table 1:** Characteristics of intervention schools in relation to all schools in the municipality and Sweden

**School**	**Age of children**	**Number of children**	**Non-Swedish background**^**§**^	**Parents with tertiary education**^**§§**^	**Prevalence overweight and obesity (10 year olds)**^**§§§**^
A	6-12	225	50%	36%	30%
B	6-12	76	NA	32%	20%
C	6-12	70	<10 students	82%	NA
D	6-12	176	<10 students	52%	9%
E	6-12	323	7%	50%	28%
F	6-16	459	6%	57%	21%
G	6-16	768	16%	47%	9%
H	6-10	213	7%	63%	NA
I	6-12	317	6%	83%	14%
All Österåker	6-16	4,610	10%	54%	21%
Sweden	6-16	991,991	18%	49%	17%^§§§§^

§ Student and/or both parents born outside of Sweden (The Swedish National Agency for Education, statistics from 2009/10); §§ At least one of the parents has more than 12 years of education (The Swedish National Agency for Education, statistics from 2009/10); §§§ Data from school health services in Österåker, children in grade 4 year 2008/09; §§§§ 7–9 year old children, according to Sjöberg et al. [8]; NA: data not available.

**Figure 1:**
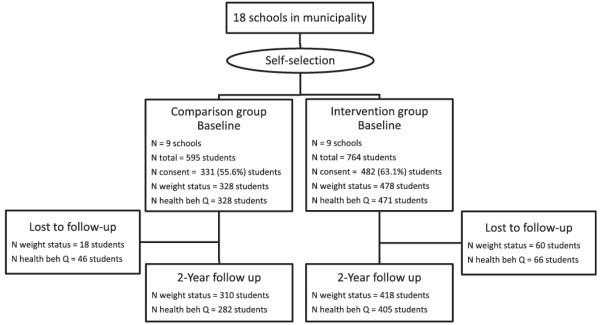
Flow diagram of recruitment and analysis.

All children in grade 2, 4 and 7 in all schools in the municipality (n=18) were invited to participate (n=1359). In total 59.8% of the children agreed to participate corresponding to 307 children in grade 2, 300 children in grade 4, and 206 children in grade 7. A flow diagram of recruitment and analysis is shown in Figure 1. Informed consent was obtained from all parents of participating children. Ethical permission for this study was obtained from the Regional Ethical Review Board in Stockholm County No. 2009/280-31/5.

### The SCIP-school programme

**Figure 2:**
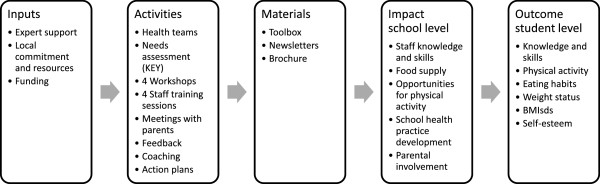
Logic model of the SCIP-school programme.

The programme is based on the social-ecological model of health targeting the individual student, the social and physical school environment and parents. The primary aim of the programme, agreed upon in the steering committee, was to improve students’ diet, physical activity and self-esteem and promote the development of healthy body weight. A programme theory was developed (Figure 2), informed by systematic reviews concerning effective school-based programmes [11,12,14,29]. Evaluation of the programme was funded by a public health fund, but no financial support was given to schools for implementation. Each intervention school was asked to form a local health team consisting of multiple professions. The health teams were invited to the first workshop in August 2008, led by the research team, and informed about the aim of the project. All workshops were documented by the research team.

The KEY to school health (KEY) is a self-assessment tool, inspired by the School Health Index, a self-assessment and planning guide [30], which helps schools to evaluate their health policies and practices with regard to strengths and weaknesses by increasing awareness and participation among school staff and to plan for action. The KEY consists of four modules (General school health practices (8 items), Physical activity (14 items), Mental health (13 items), and Nutrition (16 items)) relevant to the aims of the programme and to the Swedish school context [31]. Face validity of the KEY items was tested by repeated consultations with experts in school health promotion, physical activity, mental health, and nutrition before the study. Each item has four response categories, with the scores 0 (not in place), 1 (under development), 2 (partially in place) to 3 (fully in place).

At the first workshop in August 2008, participants of each health team were instructed to reach consensus on the appropriate scoring for their school and to fill in the KEY questionnaire (baseline), which was collected by the research team. At the end of the workshop a process evaluation with regard to usefulness of the KEY was carried out by help of a short questionnaire. Health teams requested more items in the mental health module and five more items were added making a total of 13 items. The KEY was filled in yearly (baseline, year 1 and years 2). Yearly results were expressed for each module separately and as a total score. The scores in each module from all schools were summed up and divided by the highest possible score, multiplied by 100, and expressed as a percentage.

Health teams were invited to the second workshop in September 2008 with the purpose of writing their action plans based on the KEY results. Health teams were encouraged to address at least three of the four KEY modules in their action plans and to formulate targets and strategies how to reach them. The strategies chosen consisted of a mixture of individual, group, and environmental level actions. The research team gave feed-back on all action plans, without changing the essence of the targets, and the schools developed final versions in spring 2009. The implementation of action plans was carried out by school staff, coached by the research team, from March 2009 to May 2011. During this period a toolbox containing written health education and other materials was given to each school. Four yearly newsletters were sent out to keep schools updated with the project and providing inspiration for activities.

A third workshop was arranged in April 2010 with the aim of knowledge exchange between schools. Health teams presented their action plans to each other in the form of a poster followed by discussions. Each intervention school received at least three visits by the research team during the intervention period. During 2009, all school staff was invited to four training sessions concerning health promotion, diet and health, physical activity and health, mental health, and outdoor education. Each school organised at least one meeting for parents, where a typical school meal was served, and the research team gave a presentation of the project and its background and questions were taken from the audience. All parents received a health information brochure to take home. A fourth workshop was conducted in May 2011 with decision makers, public officials from the municipality and health teams to discuss the programme’s sustainability.

### Process evaluation

Fidelity to the programme as a whole was assessed in relation to whether schools had implemented all components in the programme according to the logic model (Figure 2) and was documented by the research staff. Fidelity to school action plans was evaluated through interviews with health teams guided by a checklist in late fall 2010 as follows: The interviewer asked the health team to grade each measure in their action plan with 0=not implemented, 1=considering to implement, 2=sporadic activities have been performed, 3= full implementation achieved. The teams were also asked about number and profession of staff in the health teams. Measures included in action plans were hereafter categorised into 20 categories (Table 2).

**Table 2:** **Content of and fidelity to action plans at year 2**^**§**^

**Measures**	**Schools with measure in action plan**	**Schools with measure fully implemented year 2**	**Example of measures**
General health practices			
Knowledge, attitudes and collaboration among staff	B, D, H, I	H, I	Discussions among staff to create a common view on health
Parental involvement	A, B, D, I	A, D, I	Motivate parents to improve healthy breakfast routines
Practice development in school health services	H		Dialogue between school health care and school management on students’ health data
General health skills among students	A		Health education in classroom with homework
Physical activity			
Outdoor environment	A, B, D, E, F, I	B, D, E	Improve schoolyard by playground modification
Physical activities and play	A, B, D, E, I	A, B	Structured outdoor physical activities during school time
Outdoor education	B, D, G, I		At least one outdoor education lesson a week/month
Recess activity	E, G, D		Formulate objectives and guidelines for recess activities
Active transport to school	E	E	Walking school bus lead by parents
Appointed staff responsible for physical activity	B		Appointed staff responsible for increasing PA among students during school and leisure time
Health skills regarding PA among students	E		Health week
Collaboration with local sports clubs	A	A	Invite local sport clubs to school
Mental health			
Student self-esteem in curriculum activities	B, D, G, I	G, I	Classroom discussions on body ideal and body image
Media and gender issues in curriculum activities	B, D, H		Classroom discussions on media influence, social norms and gender roles
Empathy training for students	D, E	D, E	Revise “emotional intelligence” work plan
Knowledge, attitudes and collaboration among staff	E, H, I	E, I	Empathy training for school staff (ICDP)
Foods and meals in school			
Health skills regarding food habits among students	A, B, D, E, I	A, D, E, I	Activate students in preparing healthy snacks
Food and meal quality	E, F, H, I	E, H, I	No sweets when celebrating
Meal schedule and dining facilities	D, G	D, G	Clarify adults’ role during meal times
Knowledge, attitudes and collaboration among staff	A		Improve routines for further work with meals
Total number of measures	56	27	

§ The health teams formulated measures to be included in action plans and the researchers categorized all measures into 20 different categories shown in the table.

After the first year of intervention, all headmasters of the comparison schools were contacted once by telephone and were asked six questions concerning health promotion activities during the past year and plans for the coming year and whether they had been influenced by the project going on in the municipality in intervention schools.

### Outcome evaluation

#### Anthropometry

Height and weight were measured in a standardised way by the school nurse or by the research team. BMI was calculated (BMI = weight (kg)/height (m^2^)). Normal weight, overweight and obesity were defined using cut-off points according to the International Obesity Task Force [32]. Thinness was defined by cut-offs suggested by Cole et al. [33]. BMI standard deviation scores (BMIsds), adjusted for age and sex, were obtained based on Swedish population reference curves [34].

#### Health behaviours

A health questionnaire was sent home to all children to be answered at baseline (spring 2009) and after 2 years (spring 2011) with the help of a parent if necessary. The diet questions included frequency of breakfast, lunch, consumption of fruit, vegetables, soft drinks, candy and sweets. The answering alternatives for breakfast and lunch were on an ordinal scale with four answering alternatives: Every school day, 3–4 school days per week, 1–2 school days per week and never. The other diet questions had five answering alternatives: Two or more times per day, once per day, 3–6 times per week, 1–2 times per week and seldom. In this study the variables were dichotomised based upon current dietary recommendations [35]. The cut-points for breakfast and lunch were set at every school day, for fruit and vegetables at two or more times per day and that of sweetened drinks, candy and sweets at two or less times per week.

The physical activity assessment included five items which were likely targets for the intervention: Leisure time sports participation, time spent outdoor, active commuting, recess activity, and membership of a club. The answering alternatives are given below. For sports participation a dichotomous variable was computed with less than three times per week as cut-point [36]. For time spent outdoor an arbitrary dichotomous variable was computed with less than 30 minutes per day as the cut-point. Active commuting was reported as on how many days per week the children walked or biked to school. An arbitrary dichotomous variable was computed using less than three days per week as cut-point. Physical activity during recess was reported as on how many days they were physically activity during recess. An arbitrary dichotomous variable was computed using less than three days per week as cut-point. One question assessed whether the child was a member of a sport or other club with the answering alternative yes or no.

TV-viewing was assessed as hours in front of TV on school days and weekend days, respectively using four answering alternatives: Less than 1 hour per day, 1–3 hours per day, 3–6 hours per day and more than 6 hours per day. A dichotomous variable was computed using at most 3 hours per day as cut-point.

#### Validation of diet and physical activity questions

The diet questions were tested for validity, against a 7-day food diary in 55 fourth grade and 38 seventh grade students. The diary had to have at least four valid days to be included in the analysis. Validity of the diet questions relative to the food diary showed a value for Cohen’s weighted kappa of 0.24-0.54 in fourth grade children and 0.35-0.88 for seventh grade children, which can be considered as moderate to substantial agreement. For both age groups agreement was 69-93% for the meal pattern questions, and 31-54% for the questions about specific food items. Concerning reliability, test-retest analysis was performed three days apart and showed kappa-values of 0.17-0.59 for fourth grade and 0.65-0.96 for seventh grade students. Agreement was 74-97% for meal pattern and 41-75% for food items, which was considered satisfactory. With regard to test-retest our results in 13–14 year olds (grade 7) are comparable to those of the ENERGY questionnaire, which was tested in 11–12 year old children [37], and slightly higher than those reported by Lien et al. also for 11–12 year old children [38].

The physical activity questions were tested for validity by use of an accelerometer (Actigraph GT1M) worn for 7 days in 48 fourth grade and 38 seventh grade students. Valid days regarding accelometry were defined as at least three weekdays and one weekend day but almost all of the children had all seven valid days. First, students were categorized as low, moderate and highly activity on the basis of 3 items as follows. Each question was given 0–3 points based on the activity level with 0 as the lowest level. For leisure time sports participation students received 0 points if participating less than once per week, 1 point for 1–2 times per week, 2 points for 3–4 times per week and 3 points for 5–7 times per week. For active transport students received 0 points if not commuting actively, 1 point if commuting actively for less than 10 minutes per day, 2 points if commuting actively for at least 10 minutes per day less than 3 days per week, and 3 points if commuting actively for at least 10 minutes 3 or more times per week. For recess physical activity students received 0 points if inactive, 1 point if being active 1–2 days per week (and getting warm from activity), 2 points if active 3–4 days per week, and 3 points if being active every school day. The points from these three questions were summed and students were categorized as low activity if they had 0–2 points, moderate activity 3–6 points and high activity 7–9 points, respectively. These categories were compared with the accelerometer data categorized into three categories based on WHO recommendations (< 30 min of moderate to vigorous intensity, 30–60 min, > 60 min). The age-specific cut-points for identifying moderate and higher intensity developed by Evenson [39] and confirmed by Trost [40] were used for the accelerometer data. We found 64% agreement in the same category for fourth graders and 58% for seventh graders, which can be considered as moderate agreement. This indicates that the questionnaire can be used as a screening device to categorise children’s activity levels. As outcome measures in the intervention we calculated both the changes in the combined measure for physical activity and for each item separately.

Clustering of risk behavior was defined as having at least two of the following behaviours: Breakfast (less than every school day), lunch (less than every school day), fruits (less than twice per day), vegetables (less than twice per day), sweets (more than twice per week), soft drinks (more than twice per week), leisure time sports participation (less than three times per week) and TV-viewing (more than 3 hours per day).

#### Global self-esteem, wellbeing and dieting

Self-esteem was evaluated in grade 4 and 7 with the global self-worth subscale of Harter’s Self-Perception Profile for Adolescents [41]. The scale is a 5-item designed to assess self-esteem ranging in scores from 1 (strongly agree) to 4 (strongly disagree). The scale has been tested for validity, reliability and question format [42]. Global self-esteem was calculated by the mean of total score of all questions and categorised as very low, fairly low, fairly high and very high. For the analysis a dichotomous variable was computed using fairly high as cut-point.

Well-being was assessed using the question “This is how I feel right now” with answering alternatives bad, fairly bad, fairly good and good. A dichotomous variable was computed using fairly good as cut-point.

Dieting behavior was assessed by four questions [43]: “Have you ever tried to lose weight?”, “Do you try to lose weight today?”, “Have you ever tried to gain weight?”, and “Are you trying to gain weight now?” Answers were yes or no.

### Data analysis

Changes in KEY-scores between baseline and 1- and 2-year follow-up were assessed by Wilcoxon Signed Ranks Test, both for the total score and for separate modules. In order to identify a possible dose effect of the intervention at school level, schools were categorized into two groups according to the number of fully implemented actions, as a crude measure of intervention dose. The arbitrary cut point for low dose was set at 0–3 implemented actions and the high dose at 4–6 actions. Changes in KEY-scores were compared between the two categories by Mann–Whitney *U* Test.

Participant’s characteristics and behaviours are shown as percentages. For each grade, bivariate comparisons at baseline for the intervention and comparison group were tested using the chi-square test for categorical data, Kruskal Wallis’ test for ordinal data such as weight status, and independent samples’ *t*-test for continuous data such as BMIsds. When chi-square test was not possible to use due to small numbers, Fisher’s Exact Test was used.

The effect of the intervention was analysed first by unilevel analysis and thereafter for clustering within schools by multilevel analysis. To evaluate the effect of the intervention on health behaviours Generalized Mixed and Linear Models were used to conduct modified Poisson regressions. Relative risk (RR) and 95% confidence intervals (CI) were calculated for the behavioural outcome variables, and adjusted for gender and baseline values, using the “recommended behaviour” as the reference category. To adjust for multiple comparisons, the Holm-Bonferroni correction was applied [44]. For weight status, odds ratios (OR) and 95% CI were calculated by multinomial regression and adjusted for gender and baseline values, using “normal weight” as the reference category. Intervention effect on BMIsds was assessed for each weight strata separately, adjusted for gender and baseline values, by General Mixed and Linear models and 95% CI were calculated for the regression coefficients. A negative value means that the intervention has decreased BMIsds and a positive value that BMIsds has increased as an effect of the intervention. All analyses were conducted on the total sample and per cohort. Analysis was conducted on the complete dataset as well as using the “intention to treat” principle with imputation of missing values using the “last value carried forward” procedure [45]. A RR < 1 or OR < 1 means that the intervention was beneficial. A p-value <.05 was considered significant. Analyses were performed using the statistical program package IBM SPSS Statistics (version 20 for Windows, 2011, SPSS Inc, Chicago, IL) and Scientific Workplace (version 5.5 MacKichan Software Inc, Poulsbo, WA).

### Dropout analysis

A dropout analysis was conducted on those children who had no anthropometry data at follow-up in relation to gender, age group, weight status, and intervention group. Chi-square test was used to assess dropout differences for gender, age group and intervention group. Mann–Whitney *U* test was used to assess differences in dropout rate between weight status categories.

## Results

### Programme fidelity and action plans

Each school formed local health teams consisting of 4–11 staff. The professions involved were headmaster, school health care staff, school meal staff, physical education teachers, home economics teachers, other teachers and staff from after-school care. Health teams met every month or every other month. Programme fidelity was perfect for all schools except one (school C), which did not write an action plan. In total, twenty different categories of measures were identified in action plans shown in Table 2, including examples of typical actions performed. The interviews with health teams showed that 27 of 56 measures (48%) were fully implemented after two years. One school (F) did not succeed in implementing any measure fully.

### Impact at school level

The baseline and 1- and 2-year KEY-scores from the self-assessment of school health policies and practices are shown in Table 3. At year 1, a significant (p < .05) improvement was seen in the modules physical activity and mental health as well as in the total score, followed by a slight decline or stabilisation in year 2, with the net effect of significant improvements in the modules mental health and nutrition as well as the total score, after 2 years. Concerning a possible intervention dose effect we compared the KEY scores between 2008 and 2011 of the four most active schools (A, D, E, I) to the five least active schools (B, C, F, G, H). The most active schools showed higher improvement in the KEY modules general health practices, physical activity, and the total score, but differences were not significant.

**Table 3:** **KEY scores from the four modules and total score at baseline and after 1 and 2 years**^**§**^

**Module**	**Baseline % (range)**	**Year 1 % (range)**	**Year 2 % (range)**
General health practices	72 (56–83)	77 (48–90)	68 (38–90)
Physical activity	66 (53–83)	76 (54–95)*	72 (55–88)
Mental health	57 (44–67)^§§^	74 (64–87)*	68 (51–85)*
Meals/diet	73 (44–82)	81 (72–90)	82 (73–91)*
Total score	65 (60–78)	75 (65–90)*	72 (61–86)*

* Significant difference between baseline and follow-up at p < .05 derived from Wilcoxon signed rank test.

§ The scores in each module were summed up for each school and divided by the highest possible score for that module and then multiplied by 100. Results represent average results from all nine intervention schools; §§ Results are based on fewer questions compared with year 1 and 2.

In most comparison schools, headmasters reported that physical activity had been promoted during the period through improvements made in school yards and outdoor facilities and encouraging children to be active during leisure time. No new initiatives had been started with regard to diet and mental health. None of the interviewed headmasters thought that they had been influenced by the programme taking place in intervention schools.

### Outcome at student level

Tables 4 and 5 describe baseline characteristics of the outcome variables in the intervention and comparison group, and by cohort. The proportions of children in different weight status categories for all children combined at baseline were thinness 5.5%, normal weight 76.2%, overweight 15.1%, and obesity 3.2%. In the grade 2 cohort significantly more students in the comparison group were overweight or obese compared to the intervention group (Table 4). Significant differences between the intervention and comparison group were found with regard to eating lunch at school every school day and physical activity during recess, both variables were higher in the intervention group (Table 5). Significantly more grade 2 students in the comparison group reported eating vegetables at least twice a day and being a member of a club. In the grade 7 cohort, significantly more students in the intervention group reported consumption of sweetened drinks more than twice a week and being member of a club. No other differences were found at baseline.

**Table 4:** Descriptive data on weight status and BMIsds for intervention and comparison groups at baseline, for total sample and by cohort

	**Total**		**Grade 2 cohort**		**Grade 4 cohort**		**Grade 7 cohort**	
	**Comparison N=328**	**Intervention N=478**	**Comparison N=95**	**Intervention N=209**	**Comparison N=119**	**Intervention N=179**	**Comparison N=114**	**Intervention N=90**
Weight status								
Thinness (%)^§^	5.5	5.4	4.2	5.7*	6.7	4.5	5.3	6.7
Normal weight (%)^§§^	73.5	78.0	69.5	79.4*	73.1	76.5	77.2	77.8
Overweight (%)^§§^	17.1	13.8	21.1	12.9*	16.0	15.1	14.9	13.3
Obesity (%)^§§^	4.0	2.7	5.3	1.9*	4.2	3.9	2.6	2.2
Overweight & obesity (%)	21.0	16.5	26.3	14.8*	20.2	19.0	17.5	15.6
BMIsds (mean)	1.04	0.82	1.07	0.78	1.08	0.97	0.96	0.62

* Significant difference between intervention- and comparison group at p < .05 derived using Kruskal Wallis’ test. § Defined according to Cole [33].

§§ Defined according to Cole [32].

**Table 5:** Descriptive data on health behaviour outcome variables for intervention and comparison groups at baseline, for total sample and by cohort

	**Total**		**Grade 2 cohort**		**Grade 4 cohort**		**Grade 7 cohort**	
	**Comparison N=328 (%)**	**Intervention N=471 (%)**	**Comparison N=95 (%)**	**Intervention N=202 (%)**	**Comparison N=120 (%)**	**Intervention N=179 (%)**	**Comparison N=113 (%)**	**Intervention N=90 (%)**
Eats breakfast every school day	92.1	93.0	94.7	98.0	95.8	91.0	85.8	85.6
Eats lunch every school day	81.6	87.9*	95.7	97.5	84.2	90.4	67.0	61.1
Eats vegetables at least twice a day	36.5	32.1	47.3	33.0*	34.2	37.6	30.1	18.9
Eats fruit at least twice a day	22.4	25.5	33.3	30.7	21.7	26.3	14.2	12.2
Eats sweets at most twice a week	77.7	77.4	76.6	82.1	80.8	74.6	75.2	72.2
Drinks sweetened drinks at most twice a week	81.3	75.6	85.1	81.6	80.0	75.6	79.6	62.2**
Is member of a club	84.9	84.7	96.8	81.2***	87.5	88.8	72.3	84.4*
Sports participation at least three times a week	56.3	56.0	38.0	47.5	63.3	57.1	63.7	73.0
Spends at least 30 minutes outside every school day	87.5	89.5	94.6	92.1	88.0	90.2	80.9	82.2
Walks or bikes to school at least three days a week	73.1	70.1	59.6	59.7	81.5	79.2	75.7	75.6
Was physically active during breaks at least 3 days a week	61.4	68.4*	76.9	74.9	76.3	79.9	33.0	31.1
Watched TV at most three hours every school day	97.2	98.0	100	100	98.3	98.3	93.8	93.2
Has a fairly or very high self–esteem	97.5	98.9	98.9	99.5	99.1	98.3	94.7	98.9
Feels fairly good or good	98.2	97.9	100	99.5	98.3	97.8	96.5	94.4
Has at most one risk behaviour	21.3	20.6	30.5	23.8	18.3	22.3	16.8	10.0

* Significant difference between intervention- and comparison group at p < .05 derived using Chi-square test; ** p < .01; *** p < .001.

The dropout analysis among all students showed no gender differences. Students in the grade 2 cohort had a significantly higher dropout (13.2%) than students in the grade 4 and 7 cohort (6.7 and 8.8% respectively; p = .025). The dropout rate was higher among students with a higher weight status (overweight: 13.9% and obesity: 19.2%) than among students with lower weight status (underweight: 6.8% and normal weight: 8.6%, p = .021). Students in the intervention group had a significantly higher dropout rate than students in the control group (12.6% and 5.5% respectively; p = .001), but among dropouts there was no difference with regard to weight status between the intervention and comparison group.

All analysis were adjusted for gender and baseline values. No significant differences in intervention effect were found with regard to the health behaviour variables in the health questionnaire whether we used unilevel or multilevel analysis, therefore only results from unilevel analysis are shown. However, we found the following negative intervention effects in the cohorts: The combined variable for physical activity (p = 0.008) and physical activity during recess in the grade 4 cohort (p = 0.040), eating breakfast in the grade 7 cohort (p = 0.033), and self-esteem in the grade 7 cohort (p = 0.004). However, when using the Holm-Bonferroni correction for multiple comparisons, these changes were not significant anymore. The results did not change when using imputation of missing values using the last value carried forward procedure. We found no intervention effect on weight status either (not shown). Table 6 shows the effect of the intervention in different BMIsds strata. Again, no significant effect was found in either stratum. Dieting did not increase as a result of the intervention.

**Table 6:** **Intervention effects on different BMIsds strata for total sample and by cohort**^**§**^

	**Total**		**Grade 2 cohort**		**Grade 4 cohort**		**Grade 7 cohort**	
	**B (95% CI)**	**p**	**B (95% CI)**	**p**	**B (95% CI)**	**p**	**B (95% CI)**	**p**
BMIsds thinness^§§^	−0.79 (−1.68, 0.10)	.08	−2.14 (−6.93, 2.64)	.34	−0.13 (−0.64,0.39)	.60	−0.52 (−1.59, 0.55)	.30
BMIsds normal weight	−0.05 (−0.22, 0.12)	.58	−0.09 (−0.36, 0.18)	.50	−0.15 (−0.46,0.15)	.33	0.12 (−0.17, 0.42)	.40
BMIsds overweight & obesity^§§§^	0.26 (−0.44, 0.95)	.47	0.24 (−0.96, 1.45)	.69	0.56 (−0.41,1.52)	.25	−1.61 (−3.67, 0.45)	.12

§ All analyses are adjusted for gender and baseline values; analysis conducted using linear regression analysis in the complete dataset. §§BMIsds strata defined according to Cole et al. [33]. §§§BMIsds strata defined according to Cole et al. [32].

## Discussion

The SCIP-school project is a two-year evidence-informed health intervention in compulsory school using a participatory and flexible approach with a systematic delivery based on the literature on factors influencing successful implementation [25]. Fidelity to the programme, consisting of school self-assessment of health practices and environments, participation of staff in four workshops, four training sessions, writing of action plans and parent gatherings was almost complete, demonstrating feasibility. In contrast, fidelity to school’s own action plans was only 48%, yet positive impacts on self-reported school practices and environments, measured by the KEY, were seen in the modules physical activity, mental health and nutrition. Also, a tendency for a dose effect was found between low and high implementation schools in some of the modules. Our findings demonstrate that school staff had the will and the capacity to create their own solutions on the basis of a self-assessment and facilitation by external agents. The type and number of measures in the action plans varied, which we believe is an indication that local needs and interests were being considered, which is a success factor for implementation [25].

With regard to outcomes at student level, we could not show any improvements due to intervention with regard to diet, physical activity, self-esteem or weight status. No adverse effects on weight status, self-esteem or dieting were noted either. There might be several reasons for this apparent lack of intervention effect at student level. First, school action plans might have been insufficient due to lack of effective components. All components identified as part of effective interventions [14] were mentioned in the action plans, but not all schools included all components. Therefore, a likely explanation would be that action plans were not comprehensive enough and/or the degree of implementation was too low. On average schools fully implemented only 48% of the measures planned. In order to monitor implementation of each measure more closely, the quality and quantity ought to be assessed both with regard to the dose delivered and the dose received. In the implementation literature, a greater conceptual clarity in defining key implementation constructs has been called for [22], and we suggest that in future studies more emphasis is placed on specific and objective implementation indicators. Second, actions were not always relevant to students’ health needs. Results from the student’s questionnaires were not available at the time of writing of action plans. Therefore, the action plans probably reflected the interest of the health teams more than the needs of students, which is an important fact to consider for the future when using a participatory approach. Indeed, research has shown that programmes with a specific behavioural focus on e.g. vegetables are more effective than those addressing nutrition in general [46]. Third, the health behaviour questionnaire, which covered the past week, might not have been sensitive enough to detect changes, although it was as valid and reliable as similar questionnaires used by others [37,38]. Fourth, effects might have worn off after two years, because according to the KEY-scores, the programme was more intense at year 1. Fifth, the health behaviours of children in this middle-class municipality were already relatively good at the start of the programme and might be difficult to improve further. Compared to results in the WHO-study Health behaviours of school children, where 85% of Swedish children reported that they watched TV at most 3 hours on a week day, in the SCIP-cohort this was 93% [47]. In the Health behaviours of school children study, 80% of 13-year old children had breakfast every week day whereas in the SCIP-cohort it was 86%. Also, the obesity prevalence was around 3%, which relatively low in an international comparison. In future interventions the needs assessment should be based on local data and not just general data for the country.

Other complex community-intervention projects targeting diet and physical activity in schools with a capacity-building, multi-component and flexible approach have been more successful. The Be Active Eat Well programme from Australia [48], a quasi-experimental study, was effective in slowing the rate of weight and waist gain in children by 0.1 units in BMIsds over 3 years. This comprehensive programme used multiple intervention strategies implemented to varying degrees in different schools, as in the present study. Advantages of such approach, mentioned by the authors, are flexibility and local adaptation, promotion of sustainability, the possibility of scaling up by external funding, and can lead to local health promoting policy development and decreasing health inequalities. Another school-based 4-year intervention from the USA, used the School Health Index as a tool for self-assessment and a planning guide with a participatory approach [49] very similar to our programme and showed reductions in child obesity among disadvantaged school children, which could be enhanced by addition of community actions. In both of these countries obesity prevalence among children is 2-3-fold higher than in Sweden, which increases the chance of a favourable outcome. A similar participatory and tailored approach is recommended in Canada and is called Comprehensive School Health involving both education and changes in the school environment [50]. Using this approach in schools in socioeconomically disadvantaged areas has shown promising results with regard to healthy behaviours and obesity prevention [51]. In each school a full-time school health facilitator was placed, who coordinated all actions. This is certainly an advantage but also costly for the community.

### Lessons learnt

It seems to us and others [52] that a participatory approach based on local needs is the way forward with regard to school health promotion because it may lead to capacity-building and to potentially sustainable changes in the school environment. The will to participate in modifications to the school environment was also found in an interview study with principals and food service managers from the US [53]. On the other hand this approach presents a number of challenges to the researcher with regard to planning and evaluation by allowing for choice and local autonomy. Furthermore, we have to acknowledge that student health is one priority area among multiple competing demands in schools, the most important being academic achievement. Therefore, initiatives should be framed in terms of their potential impact on academic achievement, if possible. Greater effort should be put into buy-in of the project among all school staff, not only headmasters or health teams. This is to ensure that all participants share the same vision, which should lead to higher fidelity to local action plans and long-term support for the programme [25]. However, in spite of all our efforts action started to decline already after the first year of intervention, when the initial enthusiasm seems to have decreased suggesting that more support and guidance is needed in order to maintain the programme. Provided that extra resources can be found, we believe that the Canadian approach using school health facilitators in disadvantaged areas [51] could be a way forward also in Sweden. This would also demonstrate commitment to school health promotion at the community level, which is obviously needed for the sustainability of this work.

Second, health teams should receive stronger guidance in addressing health needs of students in their action plans, as well as on effective measures. We are currently working on a web-based system for student questionnaires, allowing a rapid feed-back of results to schools. Such local data collection and feed-back system has already been developed in Canada called SHAPES, where it has been widely disseminated [54]. Regarding the Swedish context, we believe that school health care staff must play a central role in needs assessment, because they have the mandate and the health competence to collect and analyse such data, which could be used strategically in the schools’ health promotion work. In our experience, school staff like to develop their own measures. We have to find the right balance between this desire and guidance in working evidence-based by making the scientific literature available to the staff in an adequate format (written material or lectures) and guide the writing of action plans more strongly. The need for programmes that can be embedded into school routines and which do not demand too many external resources has been emphasised [14]. School health teams should set their own goals but researchers should advise the strategies based on evidence. A way forward could be to use a stepwise approach to implementation of action plans and introduce and evaluate one component at the time, e.g. outdoor education or improved meal services, with regard to both process and outcome before introducing the next. Clearly such approach would require long-term commitment and monitoring of outcomes and randomisation of schools might not be possible.

### Strengths and limitations

The strength of our approach is that it builds on implementation and sustainability research, and is applied in a “real-world” situation. After the intervention ended interviews were performed with school staff to analyse barriers and facilitators of implementation and analysis is on-going.

There are some limitations to our study. First, all outcomes at school and student level (except for body weight and height) were self-reported and as such prone to reporting bias. Fidelity and impacts at school level, as reported by health teams in interviews and with the KEY, could therefore have been biased in favour of the intervention. However, self-evaluation is part of an effective participatory approach [25], but could be substantiated through observations in schools. Second, the design was quasi-experimental, and there is a risk of selection bias in favour of more interested schools, which could explain the positive effects seen at school level. Third, the participatory approach resulting in tailored and distinct but complex school action plans is a strength with regard to ownership and capacity-building, but also a weakness with regard to evaluation, because the measures chosen and the dose varied between schools.

## Conclusions

This model project showed a high degree of fidelity demonstrating feasibility of the SCIP-school programme with positive effects on school health practices and environments. Through ownership and capacity-building it holds the potential for sustained engagement in health promotion. In reality we noted a decreasing activity already after the first year of intervention. No significant intervention effects after two years at student level were identified for the main outcomes dietary habits, physical activity, self-esteem or body weight. The balance between the need for an effective programme with evidence-based and fully implemented action plans on the one hand and contextual relevance, building on local needs and opportunities and a participatory approach on the other, is a delicate one. We believe that in future studies, better student outcomes could probably be attained by a more focused and evidence-based approach and stepwise implementation of action plans, monitored by indicators closely matched to intervention activities. With regard to sustainability, main implementation barriers and facilitators of the SCIP-school programme will be analysed in a forthcoming study.

## Competing interests

The authors declare that there is no conflict of interest**.**

## Authors’ contributions

LSE conceived of the study, and participated in its design and coordination and drafted the manuscript. NH participated in data collection, the statistical analysis and drafting of the paper. JH designed the statistical analysis and supervised the analysis. AKQ participated in the design of the SCIP-school programme, data collection, coordination and implementation. MH participated in data analysis, interpretation of results and in drafting the manuscript. All authors read and approved the final manuscript.
